# Nasal Nanoparticle Vaccine Induces a Cross-Strain T-Cell Immunity Against *Toxoplasma gondii*

**DOI:** 10.3390/pharmaceutics18091109

**Published:** 2026-09-03

**Authors:** Aurane Lecouffe, Thomas Bouillet, Bryan Thiroux, Amélie Degraeve, Anaïs-Camille Vreulx, Romain Magnez, Angelo Scuotto, Christophe Barnier-Quer, Didier Betbeder

**Affiliations:** Vaxinano SAS, 84 Rue du Docteur Yersin, 59120 Loos, France

**Keywords:** *Toxoplasma gondii*, vaccine, nanoparticles, cross-strain immunity, intranasal immunization

## Abstract

**Background/Objectives**: *Toxoplasma gondii* is a globally distributed parasite responsible for significant morbidity in both humans and animals. VXN-Toxo, is an intranasal vaccine based on maltodextrin nanoparticles formulated with inactivated *T. gondii* parasites. A vaccination campaign conducted worldwide in zoological parks demonstrated high efficacy across multiple species and geographical regions. These findings suggest that VXN-Toxo may induce broad cross-reactive immunity against *T. gondii* strains circulating in various regions. **Methods**: To further characterize the immune mechanisms, we evaluated the cellular immune response induced by VXN-Toxo in C57BL/6 mice. Following vaccination, splenocytes were stimulated with antigens derived from multiple *T. gondii* strains, representing the major haplogroups (types I, II, III, and atypical strains). **Results**: ELISPOT analysis demonstrated that VXN-Toxo induced strong antigen-specific T cell responses, characterized by robust IFN-γ and IL-17 production upon stimulation with both homologous and heterologous antigens. Flow cytometry analysis further revealed the activation of both CD4^+^ and CD8^+^ T cells, with the notable presence of IFN-γ–producing CD8^+^ central and effector memory cells and CD4^+^ effector memory T cells. **Conclusions**: Altogether, these results indicate that VXN-Toxo induces a broad, T cell–mediated immune response with cross-reactive properties and highlight its potential as a promising vaccine candidate for both human and veterinary applications.

## 1. Introduction

Toxoplasmosis is a zoonosis caused by the parasite *Toxoplasma gondii* (*T. gondii*) [1]. The intracellular protozoan can infect most warm-blooded animals, including humans. Up to one-third of the world’s population is chronically infected through the ingestion of undercooked meat, the consumption of food or water contaminated with oocysts from cat feces, or via vertical transmission.

*T. gondii* is widely distributed worldwide and has been isolated on all continents [2,3]. In Europe and North America, the parasite population is largely composed of three clonal lineages (types I, II, and III), resulting in relatively low genetic diversity [4]. Type II strains predominate in human infections and typically cause asymptomatic or mild disease in immunocompetent individuals [5]. However, they can lead to severe complications, including miscarriage and congenital abnormalities, as well as neurological disease in immunocompromised patients. In contrast, type I and III strains are less frequent [6]. Type I strains are associated with more severe, symptomatic infections, including congenital toxoplasmosis [7], with a marked impact on the central nervous system. Conversely, type III strains are most often linked to mild or asymptomatic infections and are only rarely associated with severe clinical disease. In contrast, South America exhibits a much more diverse population structure, characterized by a predominance of atypical and recombinant strains, associated with greater genetic variability [8]. The atypical *T. gondii* strains located in this region are associated with increased virulence and severe clinical manifestations, including aggressive ocular toxoplasmosis [9] and life-threatening congenital infections.

During acute infection, immunocompetent individuals mount an effective immune response against *T. gondii*, particularly against the classical clonal lineages (types II and III), in which cell-mediated immunity plays a central role. IFN-γ-dependent pathways play a key role in controlling parasite replication. However, this immune control can be less efficient against highly virulent type I strains and atypical strains. The increased pathogenicity of these strains is associated with distinct virulence determinants and their ability to interfere with IFN-γ-mediated pathways, thereby promoting immune evasion [10,11]. Highly virulent strains such as RH (type I) can induce strong pro-inflammatory responses while simultaneously deploying immune evasion mechanisms [12,13,14,15,16], whereas atypical strains often harbor polymorphic variants of these effectors that modulate host–parasite interactions and virulence [17]. Following the acute stage, tachyzoites differentiate into bradyzoites that form intracellular cysts, primarily located within neurons and muscle cells in sites with limited immune surveillance, allowing the parasite to persist long-term despite host immunity.

The standard treatment for toxoplasmosis is currently based on sulfadiazine and pyrimethamine. Despite its efficacy against actively replicating parasites, this therapeutic approach has several important limitations, including high cost, prolonged treatment duration, and considerable toxicity [18]. Importantly, these drugs are ineffective against tissue cysts [19], allowing persistent latent infection. Furthermore, emerging drug resistance further compromises their long-term efficacy [8,18,20]. As a result, current therapeutic strategies fail to achieve complete parasite clearance and often require prolonged or repeated treatment. These limitations highlight the urgent need for the development of novel and more effective therapeutic approaches.

The most promising approach is considered to be vaccination, due to its cost-effectiveness and the benefits that a preventive strategy could provide [21]. Over the past decade, substantial efforts have been dedicated to the development of vaccine candidates against *T. gondii*. Various approaches have explored a wide range of vaccine platforms (DNA [22], live recombinant [23], subunit [24], and mRNA-based vaccines [25]), almost exclusively at the preclinical stage (murine models), with the shared objective of inducing robust cell-mediated immunity dominated by Th1-type responses. At present, no licensed vaccine is available for the prevention of toxoplasmosis in humans. In veterinary medicine, toxoplasmosis represents a major cause of reproductive failure in economically important livestock species, particularly sheep and goats. To date, only one commercially available vaccine, Ovilis^®^ Toxovax [26] (MSD Animal Health, Boxmeer, The Netherlands), based on a live attenuated *T. gondii* strain, has been approved for use in sheep. Despite its efficacy, this vaccine has several limitations, including limited stability and the potential risk of reversion to a virulent phenotype [27]. Taken together, this highlights the need for safer and more stable vaccine strategies and supports the development of alternative delivery systems, such as nasal nanoparticle-based approaches, which can enhance mucosal and systemic immune responses while improving antigen stability.

The nanoparticle-based vaccine VXN-Toxo (Vaxinano, Loos, France), composed of maltodextrin nanoparticles loaded with inactivated *T. gondii*, represents a promising and safe strategy by avoiding the limitations and risks associated with live vaccines. Delivered via the intranasal route, it promotes a Th1-biased immune response dominated by IFN-γ production [28,29], which represents a desirable vaccine approach against *T. gondii*. This vaccine has demonstrated broad efficacy across multiple species, including non-human primates [28]. Furthermore, no vaccine-associated side effects have been reported after administration in various animal species [30].

Recently, in a large-scale retrospective study, the effect of VXN-Toxo administered to captive wildlife across 20 zoos in Europe and the Americas was investigated. A total of 784 animals from 58 species (notably New World primates, lemurs, and marsupials) were included. These animals are highly susceptible to severe toxoplasmosis, likely reflecting a lack of co-evolution with *T. gondii* and an incomplete adaptation of their immune system to this parasite. Infections with classical clonal lineages (types I, II, and III) can indeed lead to severe disease, although the outcome might also be exacerbated by strain-specific virulence factors [31,32,33]. The data from this study showed a 96.7% reduction in toxoplasmosis-associated mortality following vaccination with VXN-Toxo [30]. These findings highlight the protective potential of the vaccine across multiple species and geographic regions and suggest broad efficacy against diverse *T. gondii* strains. The immune response induced by VXN-Toxo in squirrel monkeys (*Saimiri sciureus*) housed in French zoological parks has previously been investigated and showed a strong Th1-polarized cellular immune response [29]. However, due to practical and logistical challenges associated with collecting and processing biological samples from a large number of vaccinated animals distributed across multiple zoological institutions in Europe and the Americas, similar immunological analyses could not be performed in animals exposed to the genetically diverse and atypical *T. gondii* strains circulating in Latin America.

Therefore, the present study was designed to determine whether VXN-Toxo induces cross-reactive cellular immunity against genetically diverse *T. gondii* strains, which may help explain the effectiveness reported under field conditions. To this end, a murine model was used to characterize vaccine-induced cellular responses following restimulation with antigens derived from representative type I, type II, type III, and atypical *T. gondii* isolates, with particular emphasis on IFN-γ and IL-17 responses.

## 2. Materials and Methods

### 2.1. Vaccine Formulation Preparation and Characterization

#### 2.1.1. Nanoparticle Synthesis

NPLs were prepared in accordance with good manufacturing practices (GMP), as previously described [34]. Briefly, maltodextrin was dissolved in NaOH under magnetic stirring at room temperature. Epichlorohydrin (Alfa Aesar, Schiltigheim, France, cat. A15823.22) and GTMA (Glycidyltrimethylammonium chloride, Sigma-Aldrich, Saint-Quentin-Fallavier, France, cat. 50053-50ML) were then added to generate a dense cationic gel. The gel was then processed using high-pressure homogenization and ultrafiltered to eliminate residual salts. Finally, lipidation was performed with 70% (*w*/*w*) of Dipalmitoylphosphatidylglycerol (Lipoid, Les Ulis, France, cat. 560300-2150040), resulting in the final NPL preparation.

#### 2.1.2. *T. gondii* Antigen Production

*T. gondii* tachyzoites were produced in Vero cells (ATCC, Manassas, VA, USA, CCL-81) as previously described. Ethical approval was not required for the use of this commercially available established cell line. After infection and parasite amplification until host cell lysis, tachyzoites from the RH strain (ATCC, PRA-310) were harvested, purified by repeated centrifugation and DPBS washes, and inactivated by freeze–thaw cycles. These resulting antigens, hereafter referred to as “TE”, correspond to purified inactivated parasites. The other *T. gondii* strains (TgH 00001, TgH 00005, TgH 24001A, and TgH 42001A) [35], purchased from the CRB of Reims (Reims, France), were cultured using the same protocol. A BCA assay (Pierce, France) allowed the antigen dose to be expressed as total protein content.

#### 2.1.3. Vaccine Formulation

Following the inactivation of *T. gondii* tachyzoites (TE from type I strain), formulations were prepared by mixing TE and NPL at a weight ratio of 1:3 in ultrapure water (TE/NPL). The amount of antigen (TE) per dose targeted in this study was 10 µg and 30 µg of NPL per animal was administered in a total volume of 10 µL.

#### 2.1.4. Native-PAGE

The association between NPL and TE was evaluated by native polyacrylamide gel electrophoresis (Native-PAGE) under non-denaturing conditions. Free TE was able to migrate into the gel, whereas TE associated with NPL remained in the loading well and did not enter the gel matrix. A total of 3 µg of TE was loaded into each well. Following electrophoresis, gels were stained using the silver nitrate method. Band intensities were analyzed by densitometry using AzurePro software (v1.41) from a mirror image.

#### 2.1.5. Hydrodynamic Size and Surface Charge (DLS)

Formulations’ size and surface charge, as well as NPL’s and free TE’s, were characterized using dynamic light scattering (DLS) and electrophoretic light scattering (ELS) on a Zetasizer Nano ZS (Malvern, Palaiseau, France), with each sample being measured in triplicate.

#### 2.1.6. Protein Thermal Stability (NanoDSF)

The average thermal stability of all the proteins of the inactivated parasite was assessed using the Prometheus Panta system (NanoTemper Technologies, Munich, Germany). Triplicates of each sample were heated from 20 °C to 95 °C, causing proteins to progressively unfold. This unfolding was tracked through changes in the intrinsic fluorescence of tryptophan and tyrosine residues (F350nm/F330nm), allowing determination of the melting temperature (Tm). A higher Tm reflects greater thermal stability. Samples at 1 mg/mL were prepared in PBS pH 7.4 and loaded into capillaries. Data were analyzed using PR.Panta Analysis software (v1.6) (NanoTemper Technologies).

#### 2.1.7. Antigenic Analysis of Homologous and Heterogeneous (ELISA)

A comparative enzyme-linked immunosorbent assay (ELISA) was developed to evaluate the antigenic identification among TE from the different *T. gondii* strains, using the Platelia Toxo IgG kit (Biorad, Shinagawa, Tokyo, cat. 72840). Briefly, 5 µg of TE/well was coated in triplicate onto 96-well plates in carbonate buffer and incubated overnight at 4 °C. Washing and blocking steps were performed, and a Toxoplasma-positive pool serum from the kit (containing human polyclonal IgG anti-*T. gondii*) was used for the recognition of the TE components. Results were revealed using an HRP-conjugated secondary antibody and TMB substrate, then read with a spectrophotometer at 450 nm.

### 2.2. Mice

Six-week-old female C57BL/6 mice were purchased from Janvier (Paris, France). Animals were randomly placed in polypropylene cages in groups of four individuals. A temperature of 22 °C ± 2 °C and a twelve-hour light cycle (8 a.m. to 8 p.m.) were maintained. Body weights were recorded weekly, starting from the beginning of the study, and clinical signs were monitored daily. Animal procedures were carried out in accordance with the EU Directive 2010/63/EU and were approved by the local ethics committee (Genfit, Loos, France, CE077).

After a seven-day acclimatization period, immunization was performed on unanesthetized animals. First, the control group, consisting of four mice, was treated with PBS. Then, a group of eight individuals received intranasal administration of NPL/TE (30 µg of NP/dose and 10 µg of TE/dose), with a 5 µL drop placed into each nostril of the mice. Mice received two immunizations at a two-week interval. Two weeks after the final immunization, mice were anesthetized with isoflurane and sacrificed by cervical dislocation and exsanguination. No animals were excluded, as all mice remained healthy throughout the duration of the experiment. Blinding was not performed in this study; researchers were aware of group allocation during both administration and sample analysis.

### 2.3. Immune Response Analysis

#### 2.3.1. Humoral Immune Response (ELISA)

Blood samples were collected on day 0 and 28 (i.e., 14 days after the final immunization) and processed for serum isolation. Blood was centrifuged at 10,000× *g* for 10 min, and serum was subsequently collected and stored at −80 °C until further analysis.

The anti-*T. gondii* humoral response was analyzed using an indirect ELISA. Briefly, TE was coated onto 96-well plates at 5 µg per well in carbonate buffer and incubated overnight at 4 °C. The plates were washed three times with PBS-0.05% Tween20 and blocked with 200 µL of PBS-2% BSA solution for 1 h at room temperature. After an additional washing step, serum samples diluted in PBS-2% BSA (from 1:10 to 1:1280) were added and incubated for 2 h at room temperature. Following washing, 100 µL/well of HRP-conjugated goat anti-mouse IgG (ThermoFisher, Waltham, MA, USA, cat. 31430) was added and incubated for 1 h at room temperature. Plates were developed using 100 µL of TMB substrate per well (ThermoFisher, cat. N301), and the reaction was stopped using 100 µL/well of H_2_SO_4_ (1 M). Absorbance values were measured at 450 nm, and IgG titers were determined accordingly.

#### 2.3.2. Cross-Reactive Cellular Immune Response (ELISPOT)

Spleens were collected and rinsed in cold HBSS. The organs were then minced into 3–4 mm pieces in a Petri dish with fine scissors. A splenocyte suspension was obtained by crushing the organ pieces on a 70 µm strainer using a syringe piston. Cells were then rinsed with PBS, and erythrocytes were lysed using RBC Lysis Buffer (Biolegend, San Diego, CA, USA, cat. 420301) and resuspended in RPMI complete medium (RPMI 1640 supplemented with 10% SVF and 1% penicillin/streptomycin). The splenocyte suspension was used directly for ELISPOT analysis.

The cellular response was analyzed using IFN-γ and IL-17 ELISPOT kits (Mabtech, Stockholm, Sweden, cat. 3321-4APT-2, cat. 3521-4APW-2). Briefly, 1 × 10^6^ cells (at 1 × 10^7^ cells/mL) were pre-incubated in a 96-well flat-bottom plate for 24 h at 37 °C (5% CO_2_). Stimuli containing 1 µg TE/1 × 10^6^ cells of the different *T. gondii* TE were added to the cells for the pre-incubation step. After the 24 h pre-incubation step, 2 × 10^5^ cells were transferred into an ELISPOT plate. 5 µg/mL of concanavalin A was then added to the cells as a positive control, and RPMI was used as a negative control. Splenocytes were then incubated for another 24 h at 37 °C (5% CO_2_). ELISPOT plates were developed according to the manufacturer’s recommendations.

#### 2.3.3. T-Cell Phenotyping and Intracellular Cytokine Analysis (Flow Cytometry)

**Figure 1:**
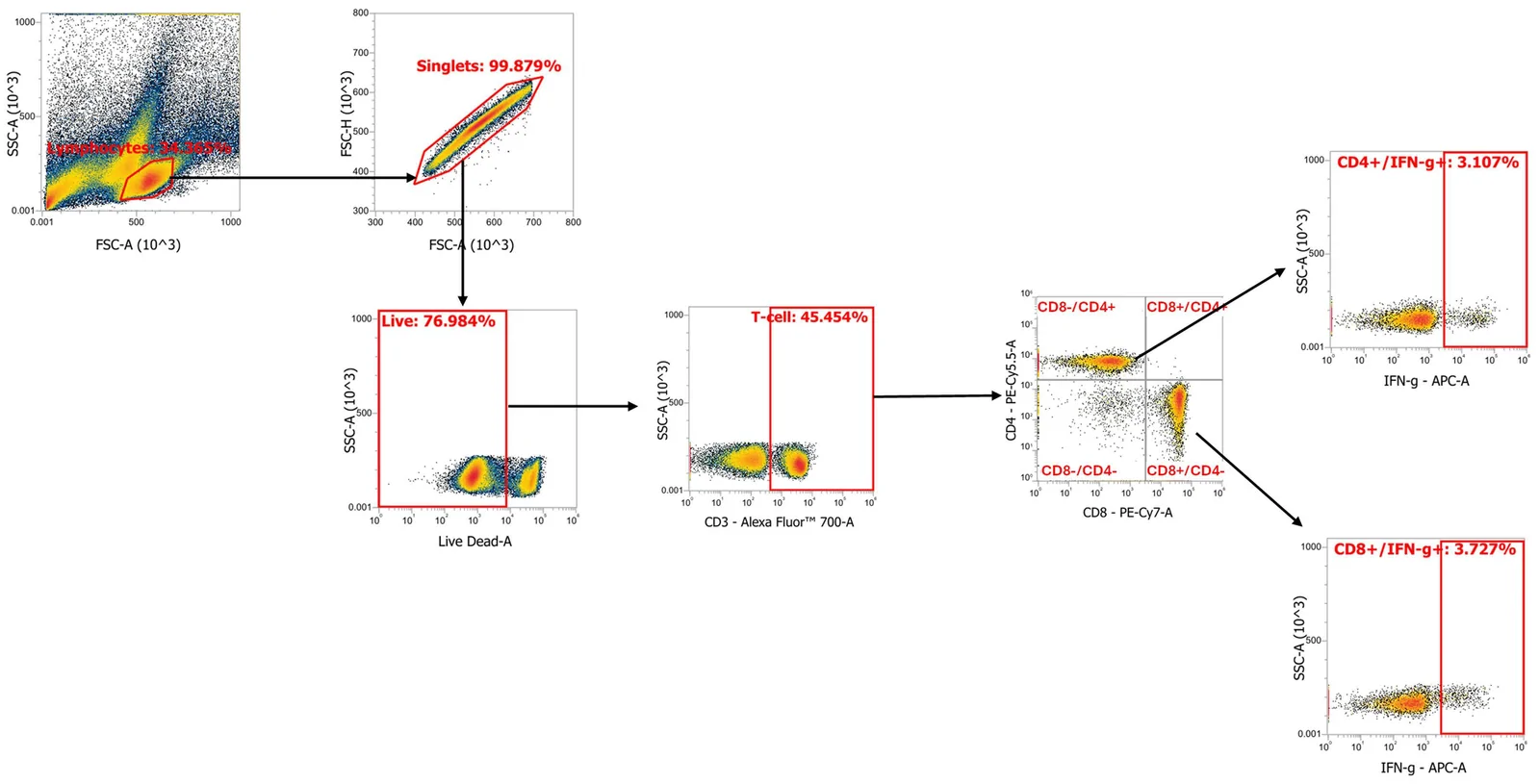
Gating strategy for flow cytometry analysis of mouse splenocytes. Example of a positive control for IFN-γ production. The lymphocyte population was identified based on FSC-A and SSC-A. Singlets were then isolated by comparing FSC-A against FSC-H. Viable cells were selected by gating on the negative population for a fixable viability dye. From the live-cell gate, total T cells were identified by CD3 expression. T cells were subsequently separated into CD4^+^ and CD8^+^ subsets using a quadrant gating strategy. Finally, intracellular IFN-γ expression was quantified within the CD4^+^ and CD8^+^ T cell subsets. Events are displayed using a pseudocolor density plot.

Splenocytes were isolated as described above, and 1 × 10^6^ cells were incubated overnight at 37 °C (5% CO_2_) with 1 µg of TE from the RH strain (Type I) or from atypical strain 2 (A2). TE I (homologous, vaccine strain) and TE A2 (heterologous, atypical strain showing the strongest IFN-γ ELISPOT cross-reactivity) were selected as representative homologous and heterologous conditions for the more resource-intensive flow cytometry analyses, given the limited cell numbers available per animal. Memory T-cell subset analysis (CD44/CD62L) was performed on cells obtained from four vaccinated mice and four non-vaccinated mice. RPMI and stimulation cocktail (ThermoFisher, cat. 00-4970-93) were used, respectively, as the negative and positive controls. Brefeldin A, a protein transport inhibitor, was added 6 h before cell collection. Cells were then collected and rinsed with PBS. Extracellular staining was then performed using the following fluorescent antibodies: Live/Dead Fixable Green (ThermoFisher, cat. L34970), CD3-AF700 (eBioscience, San Diego, CA, USA, cat. 56-0032-80), CD4-PerCP-Cyanine5.5 (eBioscience, cat. 45-0042-80), CD8a-PE-Cyanine7 (eBioscience, cat. 25-0081-81), CD44-PE (eBioscience, cat. 12-0441-81), and CD62L-APC-eFluor780 (eBioscience, cat. 47-0621-80). Intracellular staining was performed after a fixation/permeabilization step using BD Cytofix/Cytoperm (BD Bioscience, San Jose, CA, USA, cat. 554714), using an anti-mouse IFN-γ antibody (eBioscience, cat. 17-7311-82). Flow cytometry analysis was performed using an Attune Nxt cytometer (ThermoFisher) using the gating strategy described in Figure 1.

### 2.4. Statistical Analysis

The characterization data were presented as the mean ± SD. For in vivo studies, an analysis was performed using G*Power (v3.1) to determine the minimum sample size required for the study. Based on an expected effect size of 1.72, an α error probability of 0.05, and a desired power of 80%, the estimated sample size was four animals for the control group and eight animals for the vaccinated group. Data normality was assessed using the Shapiro–Wilk test. As ELISPOT spot-forming cell (SFU) counts and the percentage of IFN-γ-producing cells in flow cytometry experiments were not normally distributed, non-parametric approaches were used. Comparisons between vaccinated and unvaccinated groups were performed using the Mann–Whitney test. A *p*-value < 0.05 was selected as the statistical significance threshold. All statistical analyses were performed using GraphPad Prism version 8.4.2.

## 3. Results

### 3.1. Characterization of the Vaccine Formulation

**Figure 2:**
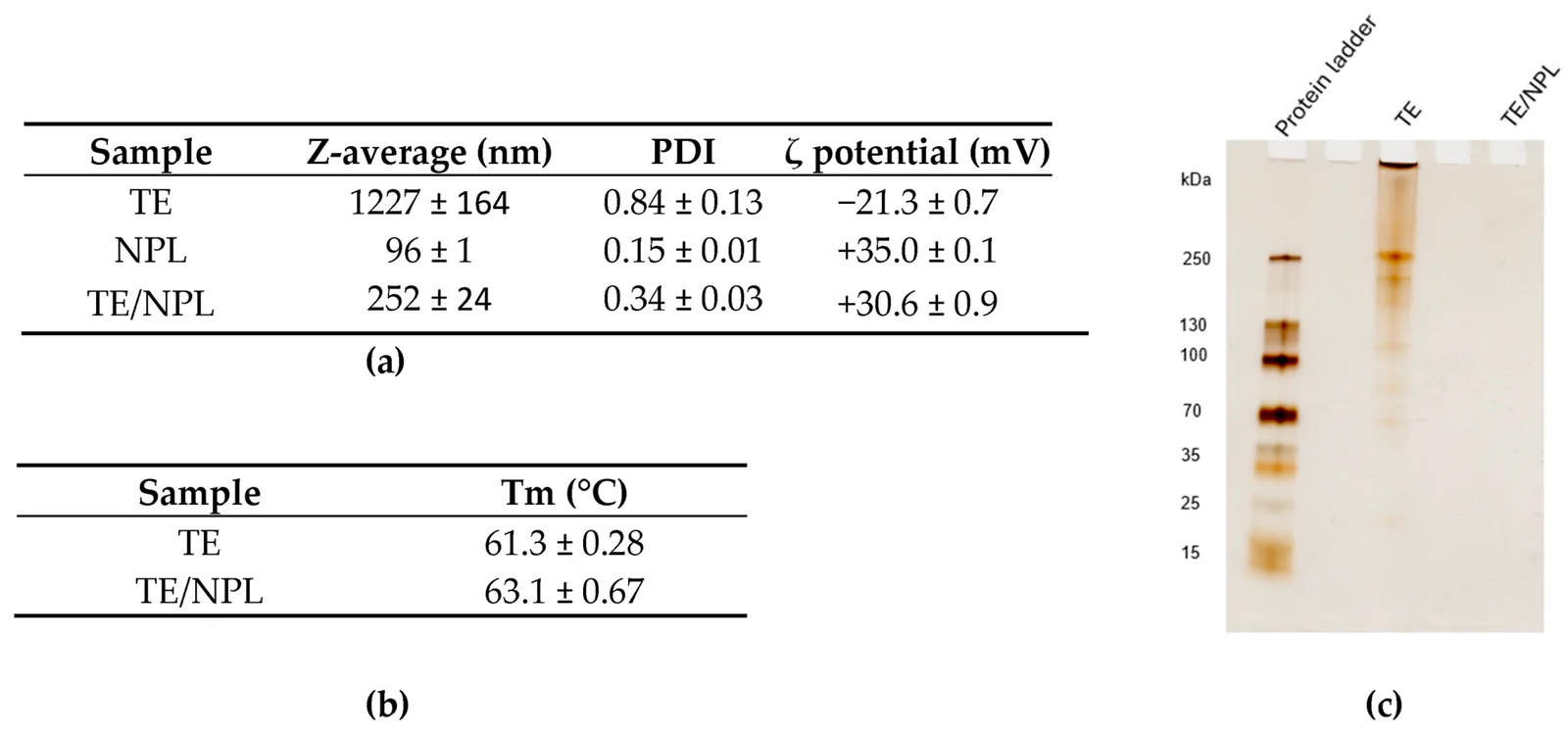
Characterization of the TE/NPL vaccine against *T. gondii.* (**a**) Table of Z-average, PDI and zeta-potentials of TE, NPL, and TE/NPL by DLS and ELS. Data are presented as mean ± SD of three replicate measurements. (**b**) Thermostability of free TE compared to TE encapsulated in NPL by NanoDSF. Data are presented as mean ± SD of three independent measurements (paired Student’s *t*-test) (**c**) Native-PAGE of the TE/NPL formulation compared to free TE. Antigen association was assessed with AzurePro software from a mirror image.

TE exhibited a hydrodynamic diameter (Z-average = 1227 nm), a polydispersity index (PDI = 0.84), and a negative surface charge (−21.3 mV), consistent with a heterogeneous size population. In contrast, NPL displayed a mean diameter (96 nm), a PDI of 0.15 and a positive surface charge (+35 mV). The association between TE and NPL was confirmed by the shift from a negative to a positive surface charge of the antigens (+30.6 mV) (Figure 2a).

Furthermore, the association of TE with NPL was demonstrated by NATIVE-PAGE (Figure 2c), showing that no free TE band was detected in the TE/NPL lane, which suggests a high degree of antigen association.

NanoDSF further confirmed the association between TE and NPL (Figure 2b). Thermal stability analysis of all the proteins of the inactivated vaccine revealed a higher Tm for TE/NPL (Tm = 63.1 ± 0.67 °C) compared to free TE (Tm = 61.3 ± 0.28 °C).

### 3.2. Characterization of the Different T. gondii Strains Following Inactivation

**Figure 3:**
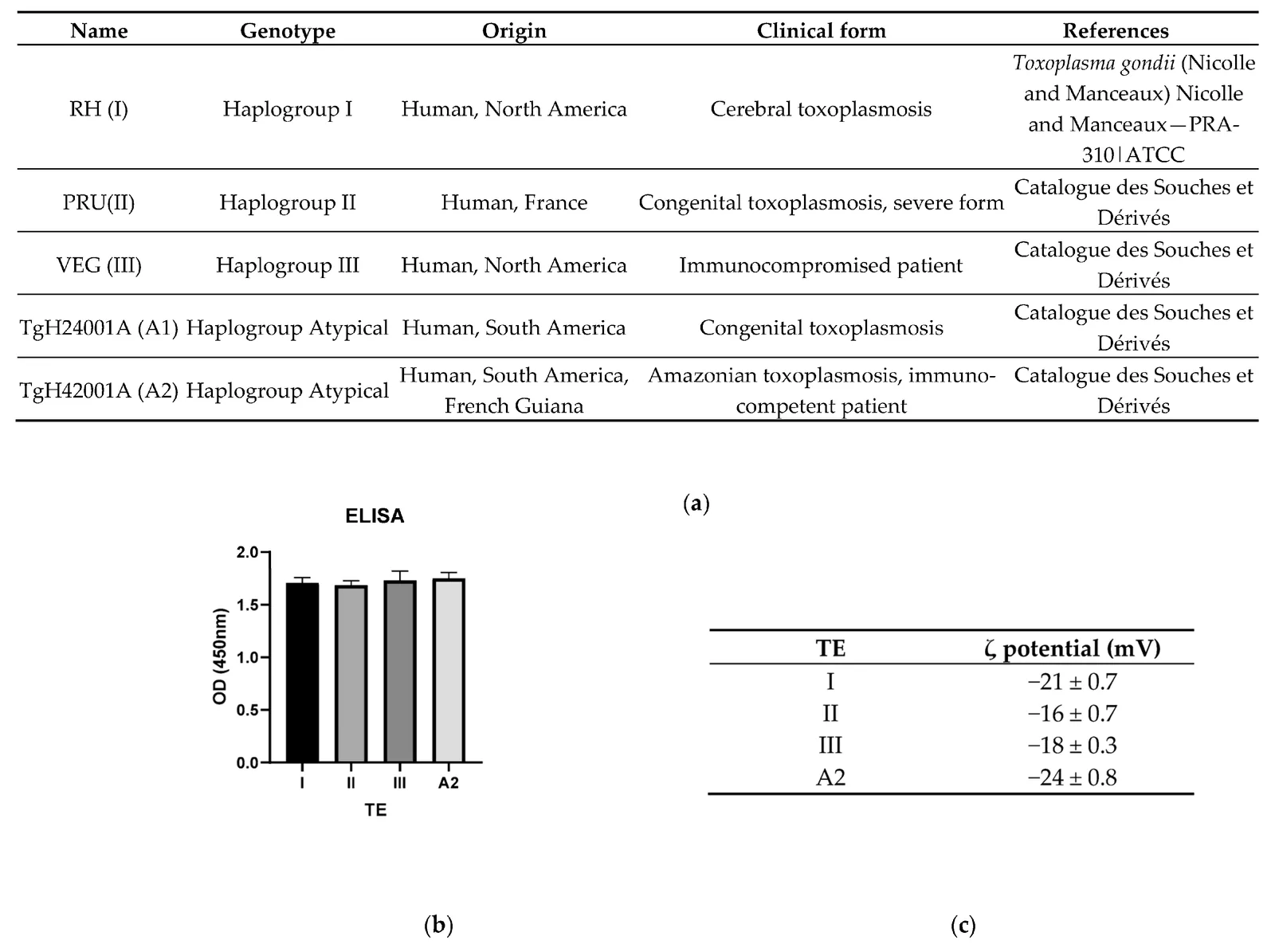
Characterization of TE from different T. gondii strains used for ex vivo stimulation of cells. (**a**) Source and genotype of the different *T. gondii* strains [35,36]. (**b**) ELISA evaluation of antigenic similarity among different *T. gondii* strains. Data are presented as mean ± SD of three replicate measurements. (**c**) Zeta potential of produced TE. Data are presented as mean ± SD of three replicate measurements.

The strains’ origins and haplogroups used to produce the different TEs are described in Figure 3a. The ELISA (Figure 3b) revealed highly comparable OD signals across the tested strains, yielding values of 1.705 for TE I, 1.685 for TE II, 1.730 for TE III, and 1.750 for TE A2. Consequently, the variation between homologous and heterologous strains was minor (<3%). This indicates that the polyclonal antibodies are able to recognize the antigens of other strains in a similar manner, demonstrating a high degree of antigenic conservation among the tested strains, but this method does not directly demonstrate the conservation of T-cell epitopes. Similarly, physicochemical characterization (Figure 3c) showed uniform, negative zeta potential values that fell within a range of −16 to −24mV, indicating comparable surface charge characteristics across the TEs.

### 3.3. TE/NPL Induces a Systemic Cellular Immune Response Against Different Strains

**Figure 4:**
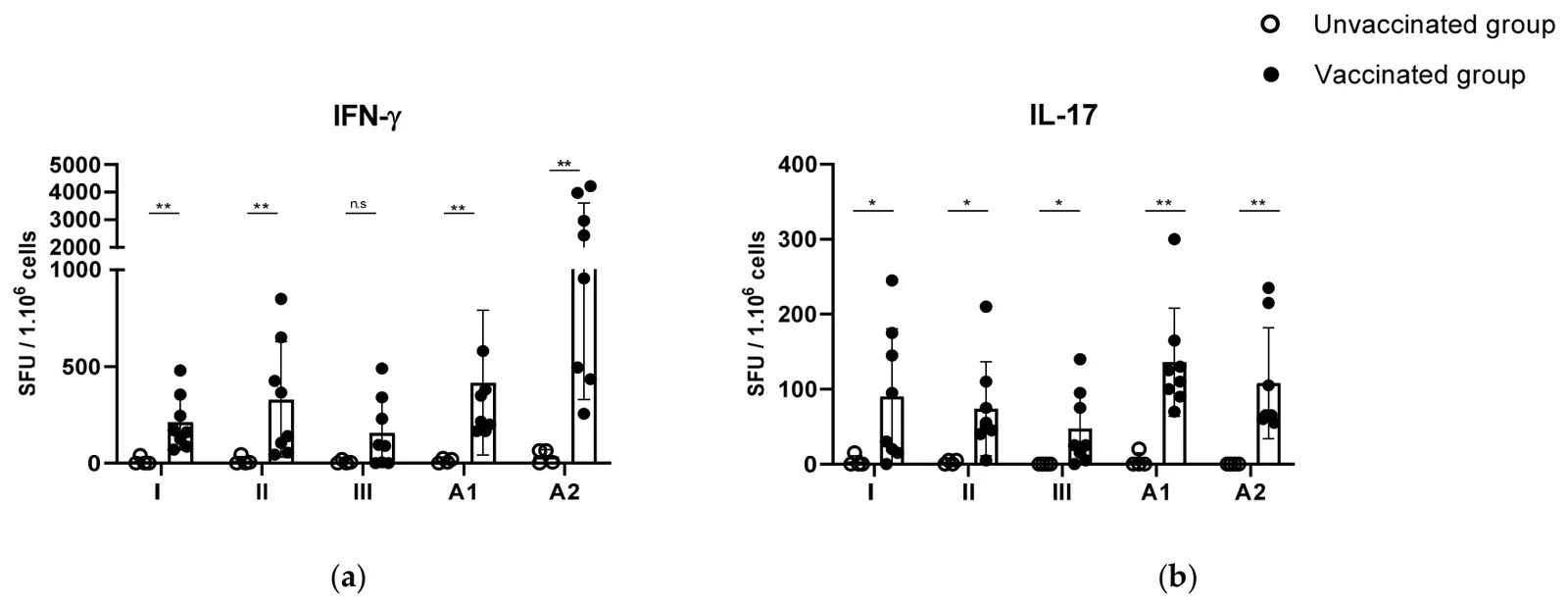
Cross-strain cellular responses induced by VXN-Toxo were assessed by ELISPOT. Splenocytes from unvaccinated (open circles, n = 4) or vaccinated (filled circles, n = 8) mice were stimulated with total extracts (TE) from *T. gondii* type I (RH), II (PRU), III (VEG), and atypical strains. (**a**) Anti-IFN-γ ELISPOT. (**b**) Anti-IL-17 ELISPOT. Each symbol represents one mouse (Mann–Whitney test, SFU: spot-forming units, ns: non-significant, *: *p*-value < 0.05, **: *p*-value < 0.01).

To determine whether VXN-Toxo induces cross-reactive immune responses, splenocytes from vaccinated and unvaccinated mice were stimulated with antigens from different *T. gondii* strains. IFN-γ and IL-17 production were then assessed by ELISPOT. As observed in Figure 4a, vaccinated mice showed a significantly higher number of IFN-γ–producing cells compared to controls following stimulation with the homologous antigen (TE I; mean = 211 vs. 10 spots). A similar trend was observed with heterologous antigens (TE II, TE A1, and TE A2), indicating the induction of cross-reactive cellular responses. Notably, stimulation with TE A2 induced particularly strong IFN-γ responses (mean = 1968 spots). In contrast, stimulation with TE III elicited IFN-γ production in only a subset of vaccinated animals (5/8), resulting in a non-significant difference compared with controls. A comparable pattern was observed for IL-17 (Figure 4b), with vaccinated mice exhibiting higher cytokine secretion than unvaccinated mice, across all tested antigens. However, unlike IFN-γ, IL-17 responses did not significantly differ in magnitude between the different stimuli. Despite notable inter-individual variability, the overall cytokine responses remained consistently higher in vaccinated animals than in controls. Finally, no significant anti-*T. gondii* IgG response was detected in the sera of vaccinated mice, with only one out of eight animals showing detectable levels of specific IgG. This result suggests that VXN-Toxo preferentially induces a cellular immune response.

### 3.4. Cellular Immune Response Is Driven by CD4^+^ and CD8^+^ T Cells

**Figure 5:**
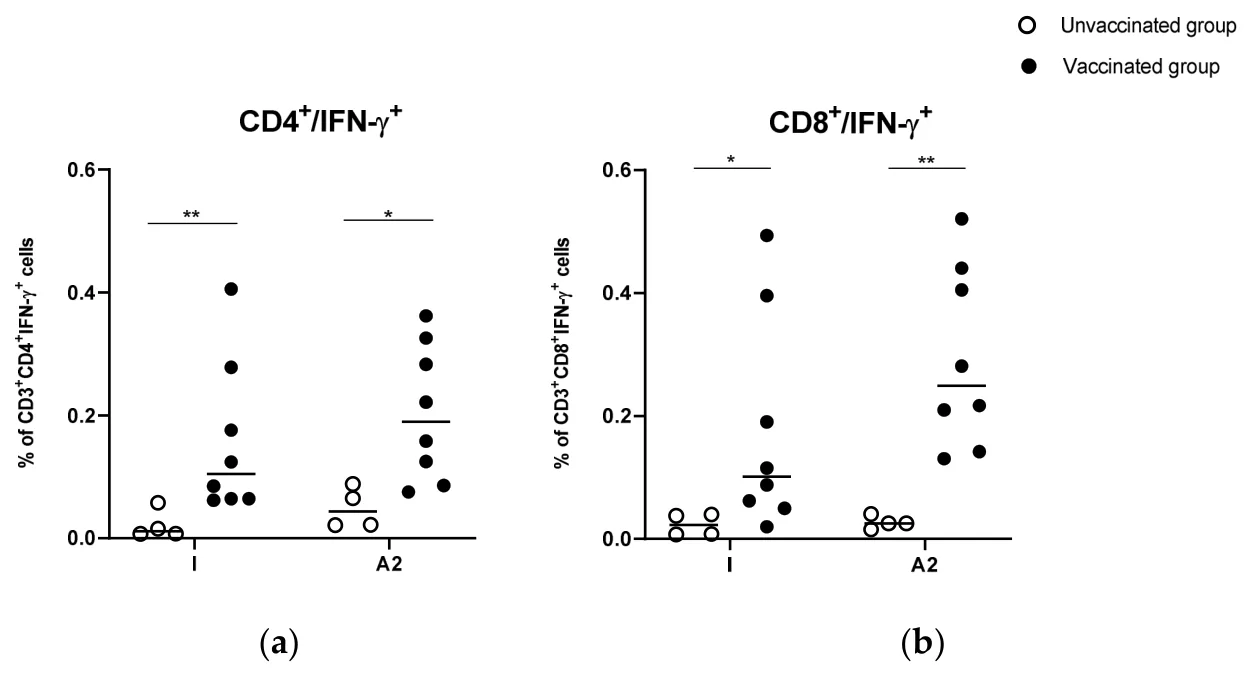
IFN-γ-producing T cells assessed by flow cytometry. Intracellular IFN-γ staining was performed on splenocytes isolated from unvaccinated (open circles, n = 4) or vaccinated (filled circles, n = 8) mice following ex vivo stimulation with TE from type I strain (I) and atypical strain (A2) of *T. gondii.* (**a**) IFN-γ production in CD4^+^ T cells. (**b**) IFN-γ production in CD8^+^ T cells. Each symbol represents one mouse (Mann–Whitney test, *: *p*-value < 0.05, **: *p*-value < 0.01).

To identify the cellular subsets contributing to the immune response, splenocytes were analyzed by flow cytometry following antigen stimulation. As shown in Figure 5, the vaccinated group displayed a significantly higher frequency of CD4^+^ T cells producing IFN-γ (median = 0.10%) compared to the unvaccinated group (median = 0.01%) upon stimulation with the homologous antigen. This response was further increased following stimulation with antigens from an atypical strain (median = 0.19%), which remained significantly elevated compared to the control group (median = 0.04%). The CD8+ T cell compartment showed a similar pattern. IFN-γ-producing cells reached 0.10% and 0.25% following stimulation with antigens from type I and atypical strains, respectively. Although no statistically significant difference was observed between A2 TE and type I TE, a trend toward a higher percentage of IFN-γ–producing cells was noted following stimulation with A2 TE, consistent with the previous ELISPOT results.

### 3.5. TE/NPL Induces Robust IFN-γ Production in Memory T Cell Subsets

To further characterize the memory T-cell response induced by VXN-Toxo, IFN-γ–producing T cells were analyzed by flow cytometry and classified into effector memory (TeM: CD44+/CD62L−) and central memory (TcM: CD44+/CD62L+) subsets based on their phenotypic markers.

**Figure 6:**
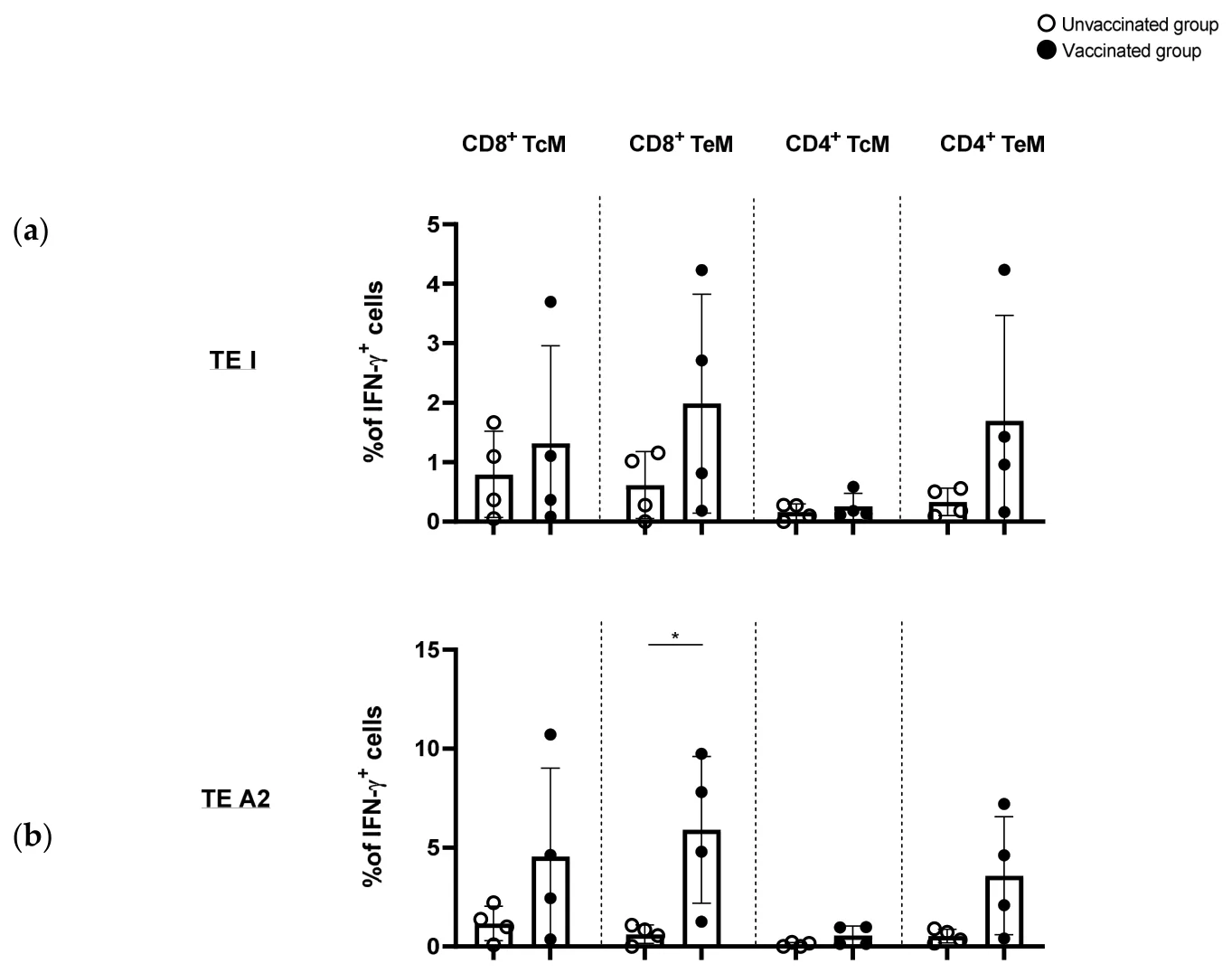
IFN-γ-producing memory T-cell subsets following stimulation assessed by flow cytometry. Intracellular IFN-γ staining was performed on splenocytes isolated from unvaccinated (open circles) or vaccinated (filled circles) mice following stimulation with TE from (**a**) type I strain and (**b**) atypical strain of *T. gondii.* Each symbol represents one mouse (Mann–Whitney test, *: *p*-value < 0.05, n = 4).

Following homologous restimulation (TE, Figure 6a), vaccinated mice displayed an overall increase in the frequency of IFN-γ–producing T cells across most subsets. This increase was particularly evident in the CD8^+^ compartment, with both TcM and TeM populations, as well as the CD4^+^ TeM subset. Upon heterologous restimulation (TE A2, Figure 6b), a similar trend was observed, with an overall stronger IFN-γ response compared to TE stimulation. Notably, the CD8^+^ TeM cells reached the highest frequencies. In contrast, in the CD4^+^ TcM compartment, IFN-γ production remained low, regardless of vaccination status or the type of restimulation used.

## 4. Discussion

### 4.1. VXN-Toxo Induces a Cross-Strain Th1/Th17 Immune Response

The present results demonstrate that intranasal vaccination with VXN-Toxo in mice induces a robust cellular immune response not only upon stimulation with homologous TE (type I) but also following exposure to heterologous *T. gondii* strains (type II, III, and atypical strains) TE. This is of particular importance given the central role of IFN-γ in host defense against *T. gondii*. IFN-γ is a key component of protective immunity, involved in both innate and adaptive immune responses. It contributes to parasite elimination by activating antimicrobial mechanisms in infected host cells, particularly macrophages [15].

Furthermore, the flow cytometry analysis showed that both CD4^+^ and CD8^+^ T-cell populations contributed to this IFN-γ production in response to homologous and heterologous antigens, highlighting their essential roles in immunity against toxoplasmosis. On the one hand, CD4^+^ T cells orchestrate the anti-parasitic immune response not only through IFN-γ secretion and macrophage activation, but also by supporting CD8^+^ T cell functions, thereby restricting parasite reactivation. On the other hand, CD8^+^ T cells complement this defense through direct cytotoxic activity and additional production of IFN-γ [37], which further activates the host cells’ autonomous clearance pathways and reinforces local parasite control. Nevertheless, the frequency of IFN-γ-producing CD4+ and CD8+ T cells observed in this study (<0.3%) is relatively low. Similar flow cytometry experiments following *T. gondii* vaccination have reported highly variable responses ranging from <0.3% to >20% (with control group values ranging between 0.1 and 15%, respectively) [38,39,40]. Overall, the differences in vaccine type, adjuvant, immunization schedule, mouse strain, and experimental design limit the interpretation and relevance of any direct comparison, and our own findings should accordingly be interpreted with caution when taken in isolation.

The increased frequency of IFN-γ-secreting central memory CD8+ T cells observed after vaccination suggests that VXN-Toxo promotes the generation of memory T cells. However, cellular immune responses were assessed at a single time point after vaccination, which is suggestive of memory induction rather than direct evidence of a long-term antigen-specific response; longitudinal follow-up would be required to confirm the persistence of this response over time [41]. The durability of the vaccine-induced immune response has been further investigated in a different animal species, seronegative squirrel monkeys (*Saimiri* spp.). Following prime immunizations and boosts at 6 and 12 months, TE-specific IFN-γ secretion by peripheral blood mononuclear cells progressively increased over time and reached its highest, statistically significant level at 12 and 24 months [29], indicating that the cellular immune response induced by VXN-Toxo can be sustained for at least two years.

Despite the increase in CD4^+^ effector memory T cells, no significant increase in CD4^+^ central memory T cells was detected in the present study. However, a previous study evaluating memory T cell subsets following ex vivo stimulation of PBMCs from Colombian patients with chronic toxoplasmosis, who were likely infected with atypical *T. gondii* strains [42], demonstrated a significant increase in IFN-γ-producing CD4^+^ central memory cells after stimulation with VXN-Toxo, particularly in patients with ocular toxoplasmosis. The absence of detectable CD4^+^ TcM responses in the present study may therefore reflect differences in species, infection history, or heterogeneity in memory marker expression compared to CD8^+^ cells [43,44].

Interestingly, the magnitude of the IFN-γ response following ex vivo stimulation showed some differences between strains, with a stronger IFN-γ response following stimulation with the atypical strains (A2). A similar trend was observed in the IFN-γ secretion of the CD4^+^ and CD8^+^ T cell subsets, and the CD8^+^ TcM and TeM populations. Despite the heterologous nature of T-cell stimulation, atypical *T. gondii* strains seem to amplify the T-cell response. Similar observations have been made in another study [45], following the in vitro stimulation of immune cells (peritoneal exudate cells) from mice previously infected with either type II or type III lineages. The data showed that restimulation with the atypical MAS strain consistently yielded higher frequencies of IFN-γ-producing CD8^+^ T cells. This might reflect the presence of conserved or more immunogenic epitopes that are more efficiently recognized by vaccine-induced T cells, leading to increased T-cell activation despite the different strain used by the vaccine (type I). Marked interindividual variability in cytokine responses, previously reported during natural *T. gondii* infection [46], likely reflects the inherent biological heterogeneity of cellular immune responses, as well as technical variability associated with ex vivo cytokine assays.

The results of this study also indicate that IL-17 production was detected in splenocytes after restimulation with both homologous and heterologous strains. IL-17 is known to contribute to early protective immunity against *T. gondii*, particularly by promoting the recruitment of neutrophils to sites of infection [47]. Similarly, vaccination with VXN-Toxo in mice significantly increased splenocyte secretion of IFN-γ, TNF-α, IL-6, and IL-17 [48], and a comparable Th1/Th17-polarized signature has also been documented in non-human primates [49], suggesting that this profile plays an essential role in the effective control of *T. gondii* infection.

### 4.2. VXN-Toxo Immunogenicity: Role of Formulation and Route of Administration

The physicochemical characterization of the formulation provides further insight into the interaction between the antigen and NPL. NanoDSF analysis showed a modest but detectable thermal shift following formulation. This limited change may indicate that the overall structure of the antigen mixture remains largely preserved upon formulation with NPL [50]. Further stability studies would be required to determine whether this effect improves antigen preservation during storage or has an impact on vaccine performance.

The change in particle size and surface charge after formulation supports the formation of TE/NPL complexes through electrostatic interactions between the anionic TE antigens and the cationic NPL nanoparticles. The immunological relevance of this association was previously investigated [48,51], as free TE and empty NPL failed to induce cytokine secretion and to confer protection against *T. gondii* challenge, whereas immunization with VXN-Toxo (TE/NPL) conferred full survival and a significant reduction in brain cyst burden. These findings indicate the dual role of NPL in both antigen delivery and immune potentiation.

As previously reported, the intranasal route of administration likely plays a key role by targeting the nasal-associated lymphoid tissue (NALT) [52,53]. This mucosal delivery is efficiently mediated by NPL [54], facilitates antigen delivery in the presence of mucins to antigen-presenting cells (APCs), and activates Toll-like receptor signaling pathways (TLR2 and TLR4) [55]. The activation of these pathways plays an essential role in the development of a Th1/Th17 immune response [56] and the induction of both systemic and mucosal immunity [57,58].

In contrast, the VXN-Toxo vaccine did not elicit a significant humoral immune response after two immunizations. This is consistent with previous results showing a poor humoral immune response [29]. Therefore, the main hypothesis for the protective efficacy of VXN-Toxo was attributed to the cellular response. In natural immunity, the humoral response also plays a relatively minor role in protection against *T. gondii* infection [56,59].

### 4.3. Toward Broadly Cross-Reactive and Cross-Protective Toxoplasmosis Vaccines

*T. gondii* strains are classically grouped into three major clonal lineages (types I, II, and III) as well as a wide range of atypical genotypes, based on multilocus genotyping using polymorphic markers [8]. Atypical strains display increased genetic diversity, largely driven by recombination events. However, despite this diversity, whole-genome analyses have revealed a high level of overall genetic conservation among *T. gondii* strains, with more than 99% sequence identity. Importantly, the limited genetic variation is concentrated within a restricted number of polymorphic loci, particularly those encoding secreted virulence factors such as ROP18, ROP5, and GRA15 [60]. These polymorphic effectors are sufficient to generate major differences in pathogenicity, as they directly interfere with host intracellular defense mechanisms, including IFN-γ-mediated pathways, and modulate key signaling cascades such as NF-κß and STAT pathways [61,62]. In this context, the ability to induce a broadly cross-reactive immune response appears critical.

Subunit and epitope-based vaccines, targeting immunodominant antigens such as surface antigens [24] (SAGs), dense granule [63] (GRA) proteins, and rhoptry (ROP) proteins [23], often require adjuvants and generally provide only partial and strain-restricted protection. This limitation is likely due either to antigenic variability among strains or to the lack of a strong cellular immune response induced by the vaccine [64,65]. In contrast, recent studies using live-attenuated vaccines derived from type I strains (e.g., RHΔNPT1, RHΔompdcΔuprt, RHΔhad2a) have demonstrated that they can induce broad cross-protective immunity against multiple *T. gondii* strains [66,67]. These vaccines also induce a strong Th1-biased response, characterized by high IFN-γ production and activation of CD8^+^ T cells, and are thought to mimic the natural immunity observed following primary infection, which can confer protection against secondary infection with heterologous strains. However, despite their efficacy, live-attenuated vaccines still raise important safety and regulatory concerns, including the risk of reversion to virulence, which is a major barrier to their use in humans and immunocompromised hosts.

The broad cellular response induced by VXN-Toxo may be driven by the presentation of multiple epitopes derived from the whole inactivated RH strain. This multiepitope exposure, rather than reliance on a single dominant antigen, likely promotes cross-recognition of diverse *T. gondii* strains. The strong T-cell-mediated responses observed in this study are consistent with this hypothesis, reflecting cross-reactive recognition of epitopes conserved across strains. Such an approach may represent a valuable strategy for overcoming the challenges associated with *T. gondii* genetic diversity while minimizing the risks inherent to live vaccines.

Given this cross-reactive cellular immunity and the safety profile typical of an inactivated vaccine, VXN-Toxo appears to be a promising vaccine candidate. VXN-Toxo has already been evaluated in homologous and heterologous challenge models in both mice [48] and sheep [68]. In the murine model, VXN-Toxo conferred 100% survival against a lethal heterologous challenge and a 70% reduction in cerebral cyst burden compared to unvaccinated controls. Vaccinated sheep were fully protected against cerebral cyst formation following heterologous challenge (type II Me49 strain). Similarly, vaccination of non-human primates in zoological collections was associated with a 96.7% reduction in toxoplasmosis-related mortality under natural exposure conditions [30]. Together, these studies provide strong evidence of VXN-Toxo’s efficacy. Further studies involving additional haplogroups and genetically diverse isolates are nonetheless warranted.

Beyond its prophylactic potential, the vaccination study in zoological parks suggests that VXN-Toxo may also exert beneficial effects in previously infected individuals [30]. In this study, the vaccination appeared to delay disease progression and reduce mortality in seropositive animals, suggesting a possible reactivation of pre-existing T-cell responses by the vaccine. The maltodextrin nanoparticles have already demonstrated their efficacy as a therapeutic vaccine platform against *Leishmania* [69]. Therefore, the robust Th1 response and the activation of CD4^+^ and CD8^+^ T-lymphocyte subsets observed in this study raise the possibility that VXN-Toxo could also be explored as a therapeutic vaccine strategy for patients with chronic or recurrent *T. gondii* infections.

## 5. Conclusions

The present study demonstrates that VXN-Toxo induces robust, antigen-specific immune responses against a broad range of *T. gondii* strains representing the major haplogroups. The immune response elicited by VXN-Toxo was mainly driven by cellular immunity, characterized by IFN-γ production from both CD4^+^ and CD8^+^ T-cell subsets. These findings indicate that VXN-Toxo induces a broad, cross-reactive cellular immune response against genetically diverse parasite strains, consistent with a possible immunological mechanism underlying the field effectiveness previously reported in other vaccinated animal species.

## Data Availability

The original contributions presented in this study are included in the article. Further inquiries can be directed to the corresponding author. Protocol and registration: A study protocol detailing the research question, study design, and analysis plan was developed before the study commenced. The protocol was not formally registered.

## Abbreviations

The following abbreviations are used in this manuscript:

APCs	Antigen-presenting cells
CRB	Biological Resource Center
ELISA	Enzyme-Linked Immunosorbent Assay
ELISPOT	Enzyme-Linked Immunospot
EU	European Union
IFN-γ	Interferon-gamma
IL-17	Interleukin-17
nanoDSF	Nano-Differential Scanning Fluorimetry
NPL	Lipid core maltodextrin nanoparticles
*T. gondii*	*Toxoplasma gondii*
TcM	Central Memory T cell
TE	Pure inactivated parasites
TeM	Effector Memory T cell
Th1	T helper 1
Th17	T helper 17
Tm	Melting temperature

## References

[1] de Barros R.A.M., Torrecilhas A.C., Marciano M.A.M., Mazuz M.L., Pereira-Chioccola V.L., Fux B. (2022). Toxoplasmosis in Human and Animals Around the World. Diagnosis and Perspectives in the One Health Approach. Acta Trop..

[2] Shwab E.K., Zhu X.Q., Majumdar D., Pena H.F.J., Gennari S.M., Dubey J.P., Su C. (2014). Geographical patterns of *Toxoplasma gondii* genetic diversity revealed by multilocus PCR-RFLP genotyping. Parasitology.

[3] Hosseini S.A., Amouei A., Sharif M., Sarvi S., Galal L., Javidnia J., Pagheh A.S., Gholami S., Mizani A., Daryani A. (2019). Human toxoplasmosis: A systematic review for genetic diversity of *Toxoplasma gondii* in clinical samples. Epidemiol. Infect..

[4] Fernández-Escobar M., Schares G., Maksimov P., Joeres M., Ortega-Mora L.M., Calero-Bernal R. (2022). *Toxoplasma gondii* Genotyping: A Closer Look Into Europe. Front. Cell. Infect. Microbiol..

[5] Robert-Gangneux F., Dardé M.-L. (2012). Epidemiology of and Diagnostic Strategies for Toxoplasmosis. Clin. Microbiol. Rev..

[6] Barbosa I.M.F.N., Do Prado D.P.G., Costa W.L.G., Domann N., Rezende S.R., Bridi V., de Moura V.O.L., Rezende H.H.A. (2023). *Toxoplasma gondii* Genotypes Isolated from Humans: A Systematic Review. Contrib. LAS Cienc. Soc..

[7] Xiao J., Yolken R.H. (2015). Strain hypothesis of *Toxoplasma gondii* infection on the outcome of human diseases. Acta Physiol..

[8] Brito R.M.D.M., De Lima Bessa G., Bastilho A.L., Dantas-Torres F., de Andrade-Neto V.F., Bueno L.L., Fujiwara R.T., Magalhães L.M.D. (2023). Genetic diversity of *Toxoplasma gondii* in South America: Occurrence, immunity, and fate of infection. Parasit. Vectors.

[9] Greigert V., Bittich-Fahmi F., Pfaff A.W. (2020). Pathophysiology of ocular toxoplasmosis: Facts and open questions. PLoS Neglected Trop. Dis..

[10] Moghaddami R., Moradi K., Mahdipour M., Pagheh A.S., Razeghi J., Yamchi N.N., Khordadmehr M., Nematollahi A., Ghorbanzadeh B., Ahmadpour E. (2025). Strain-dependent effects of *Toxoplasma gondii* on ovarian health and inflammation in a rat model. BMC Infect. Dis..

[11] Angel S.O., Vanagas L., Alonso A.M. (2024). Mechanisms of adaptation and evolution in *Toxoplasma gondii*. Mol. Biochem. Parasitol..

[12] Chiebao D.P., Bartley P.M., Chianini F., Black L.E., Burrells A., Pena H.F.J., Soares R.M., Innes E.A., Katzer F. (2021). Early immune responses and parasite tissue distribution in mice experimentally infected with oocysts of either archetypal or non-archetypal genotypes of *Toxoplasma gondii*. Parasitology.

[13] Zhang Y., Jiang N., Zhang T., Wang D., Feng Y., Sang X., Yang N., Chen Q. (2018). *Toxoplasma gondii* Genotype Determines Tim-3 Expression Levels in Splenic and Circulatory T Cells in Mice. Front. Microbiol..

[14] Nyonda M.A., Hammoudi P.M., Ye S., Maire J., Marq J., Yamamoto M., Soldati-Favre D. (2021). *Toxoplasma gondii* GRA60 is an effector protein that modulates host cell autonomous immunity and contributes to virulence. Cell. Microbiol..

[15] Lüder C.G.K. (2024). IFNs in host defence and parasite immune evasion during *Toxoplasma gondii* infections. Front. Immunol..

[16] Chen M., Yao L., Zhou L., Yang P., Zou W., Xu L., Li S., Peng H. (2022). *Toxoplasma gondii* ROP18I inhibits host innate immunity through cGAS-STING signaling. FASEB J..

[17] Murillo-León M., Müller U.B., Zimmermann I., Singh S., Widdershooven P., Campos C., Alvarez C., Könen-Waisman S., Lukes N., Ruzsics Z. (2019). Molecular mechanism for the control of virulent *Toxoplasma gondii* infections in wild-derived mice. Nat. Commun..

[18] Montazeri M., Sharif M., Sarvi S., Mehrzadi S., Ahmadpour E., Daryani A. (2017). A Systematic Review of In vitro and In vivo Activities of Anti-Toxoplasma Drugs and Compounds (2006–2016). Front. Microbiol..

[19] Hajj R.E., Tawk L., Itani S., Hamie M., Ezzeddine J., El Sabban M., El Hajj H. (2021). Toxoplasmosis: Current and Emerging Parasite Druggable Targets. Microorganisms.

[20] De Lima Bessa G., De Almeida Vitor R.W., Dos Santos Martins-Duarte E. (2021). *Toxoplasma gondii* in South America: A differentiated pattern of spread, population structure and clinical manifestations. Parasitol. Res..

[21] Chu K.B., Quan F.S. (2021). Advances in *Toxoplasma gondii* Vaccines: Current Strategies and Challenges for Vaccine Development. Vaccines.

[22] Zheng B., Ding J., Lou D., Tong Q., Zhuo X., Ding H., Kong Q., Lu S. (2019). The Virulence-Related MYR1 Protein of *Toxoplasma gondii* as a Novel DNA Vaccine Against Toxoplasmosis in Mice. Front. Microbiol..

[23] Grzybowski M.M., Dziadek B., Gatkowska J.M., Dzitko K., Długońska H. (2015). Towards vaccine against toxoplasmosis: Evaluation of the immunogenic and protective activity of recombinant ROP5 and ROP18 *Toxoplasma gondii* proteins. Parasitol. Res..

[24] Zhang D., Jiang N., Chen Q. (2019). Vaccination with recombinant adenoviruses expressing *Toxoplasma gondii* MIC3, ROP9, and SAG2 provide protective immunity against acute toxoplasmosis in mice. Vaccine.

[25] Li D., Zhang Y., Li S., Zheng B. (2023). A novel *Toxoplasma gondii* TGGT1_316290 mRNA-LNP vaccine elicits protective immune response against toxoplasmosis in mice. Front. Microbiol..

[26] Hasan T., Nishikawa Y. (2022). Advances in vaccine development and the immune response against toxoplasmosis in sheep and goats. Front. Vet. Sci..

[27] Innes E.A., Bartley P.M., Rocchi M., Benavidas-Silvan J., Burrells A., Hotchkiss E., Chianini F., Canton G., Katzer F. (2011). Developing vaccines to control protozoan parasites in ruminants: Dead or alive?. Vet. Parasitol..

[28] Fasquelle F., Scuotto A., Vreulx A.C., Petit T., Charpentier T., Betbeder D. (2023). Nasal vaccination of six squirrel monkeys (*Saimiri sciureus*): Improved immunization protocol against *Toxoplasma gondii* with a nanoparticle-born vaccine. Int. J. Parasitol. Parasites Wildl..

[29] Ducournau C., Cantin P., Alerte V., Quintard B., Popelin-Wedlarski F., Wedlarski R., Ollivet-Courtois F., Maltot J.F.-P., Herkt C., Fasquelle F. (2023). Vaccination of squirrel monkeys (*Saimiri* spp.) with nanoparticle-based *Toxoplasma gondii* antigens: New hope for captive susceptible species. Int. J. Parasitol..

[30] Scuotto A., Ogonczyk-Makowska D., Quiévy A., Berthet M., Schlax K., Boussarie D., Maillot A., Popelin-Wedlarski F., Charpentier T., Perot M. (2025). Retrospective Analysis of the Impact of Vaccination with an Inactivated Vaccine on Toxoplasmosis-Associated Mortality in Captive Wildlife. Vaccines.

[31] Carme B., Ajzenberg D., Demar M., Simon S., Dardé M.L., Maubert B., de Thoisy B. (2009). Outbreaks of toxoplasmosis in a captive breeding colony of squirrel monkeys. Vet. Parasitol..

[32] Salant H., Weingram T., Spira D.T., Eizenberg T. (2009). An outbreak of Toxoplasmosis amongst squirrel monkeys in an Israeli monkey colony. Vet. Parasitol..

[33] Cedillo-Peláez C., Rico-Torres C.P., Salas-Garrido C.G., Correa D. (2011). Acute toxoplasmosis in squirrel monkeys (*Saimiri sciureus*) in Mexico. Vet. Parasitol..

[34] Paillard A., Passirani C., Saulnier P., Kroubi M., Garcion E., Benoît J.-P., Betbeder D. (2010). Positively-Charged, Porous, Polysaccharide Nanoparticles Loaded with Anionic Molecules Behave as ‘Stealth’ Cationic Nanocarriers. Pharm. Res..

[35] Catalogue des Souches et Dérivés. https://www.toxocrb.com/catalogue-des-souches-2/.

[36] *Toxoplasma gondii* (Nicolle and Manceaux) Nicolle and Manceaux—PRA-310|ATCC. https://www.atcc.org/products/pra-310.

[37] Li J., Gao W., Yan Z., Yan B., Zhang J. (2025). Cellular immune response during *Toxoplasma gondii* infection: Deciphering diverse population immune variations. Trends Parasitol..

[38] Karakavuk M., Can H., Gül A., Döşkaya A.D., Alak S.E., Ün C., Gürüz A.Y., Döşkaya M. (2021). GRA8 DNA vaccine formulations protect against chronic toxoplasmosis. Microb. Pathog..

[39] Xu B., Bi Y., Wang Y., Sun J., Chen J., Mu J. (2025). Evaluation of immune protection of a multi-antigenic DNA vaccine encoding TgROP6 and TgMIC12 against *Toxoplasma gondii* infection. Front. Vet. Sci..

[40] Şahar E.A., Can H., İz S.G., Döşkaya A.D., Kalantari-Dehaghi M., Deveci R., Gürüz A.Y., Döşkaya M. (2020). Development of a hexavalent recombinant protein vaccine adjuvanted with Montanide ISA 50 V and determination of its protective efficacy against acute toxoplasmosis. BMC Infect. Dis..

[41] El Bissati K., Krishack P.A., Zhou Y., Weber C.R., Lykins J., Jankovic D., Edelblum K.L., Fraczek L., Grover H., Chentoufi A.A. (2023). CD4+ T Cell Responses to *Toxoplasma gondii* Are a Double-Edged Sword. Vaccines.

[42] García-López L., Zamora-Vélez A., Vargas-Montes M., Sanchez-Arcila J.C., Fasquelle F., Betbeder D., Gómez-Marín J.E. (2024). Human T-cell activation with *Toxoplasma gondii* antigens loaded in maltodextrin nanoparticles. Life Sci. Alliance.

[43] Natalini A., Simonetti S., Favaretto G., Peruzzi G., Antonangeli F., Santoni A., Muñoz-Ruiz M., Hayday A., Di Rosa F. (2021). OMIP -079: Cell cycle of CD4^+^ and CD8^+^ naïve/memory T cell subsets, and of Treg cells from mouse spleen. Cytom. A.

[44] Sckisel G.D., Mirsoian A., Minnar C.M., Crittenden M., Curti B., Chen J.Q., Blazar B.R., Borowsky A.D., Monjazeb A.M., Murphy W.J. (2017). Differential phenotypes of memory CD4 and CD8 T cells in the spleen and peripheral tissues following immunostimulatory therapy. J. Immunother. Cancer.

[45] Jensen K.D.C., Camejo A., Melo M.B., Cordeiro C., Julien L., Grotenbreg G.M., Frickel E.-M., Ploegh H.L., Young L., Saeij J.P.J. (2015). *Toxoplasma gondii* Superinfection and Virulence during Secondary Infection Correlate with the Exact ROP5/ROP18 Allelic Combination. mBio.

[46] Maecker H.T., Rinfret A., D’Souza P., Darden J., Roig E., Landry C., Hayes P., Birungi J., Anzala O., Garcia M. (2005). Standardization of cytokine flow cytometry assays. BMC Immunol..

[47] Alizadeh P., Ahmadpour E., Daryani A., Kazemi T., Spotin A., Mahami-Oskouei M., Flynn R.J., Azadi Y., Rajabi S., Sandoghchian S. (2019). IL-17 and IL-22 elicited by a DNA vaccine encoding ROP13 associated with protection against *Toxoplasma gondii* in BALB/c mice. J. Cell. Physiol..

[48] Dimier-Poisson I., Carpentier R., N’Guyen T.T.L., Dahmani F., Ducournau C., Betbeder D. (2015). Porous nanoparticles as delivery system of complex antigens for an effective vaccine against acute and chronic *Toxoplasma gondii* infection. Biomaterials.

[49] Kelly M.N., Kolls J.K., Happel K., Schwartzman J.D., Schwarzenberger P., Combe C., Moretto M., Khan I.A. (2005). Interleukin-17/Interleukin-17 Receptor-Mediated Signaling Is Important for Generation of an Optimal Polymorphonuclear Response against *Toxoplasma gondii* Infection. Infect. Immun..

[50] Lisina S., Inam W., Huhtala M., Howaili F., Zhang H., Rosenholm J.M. (2023). Nano Differential Scanning Fluorimetry as a Rapid Stability Assessment Tool in the Nanoformulation of Proteins. Pharmaceutics.

[51] Fasquelle F., Dubuquoy L., Betbeder D. (2022). Starch-based NP act as antigen delivery systems without immunomodulating effect. PLoS ONE.

[52] Kehagia E., Papakyriakopoulou P., Valsami G. (2023). Advances in intranasal vaccine delivery: A promising non-invasive route of immunization. Vaccine.

[53] Marasini N., Skwarczynski M., Toth I. (2017). Intranasal Delivery of Nanoparticle-Based Vaccines. Ther. Deliv..

[54] Fasquelle F., Carpentier R., Demouveaux B., Desseyn J.L., Betbeder D. (2020). Importance of the Phospholipid Core for Mucin Hydrogel Penetration and Mucosal Cell Uptake of Maltodextrin Nanoparticles. ACS Appl. Bio Mater..

[55] Vargas-Montes M., Fasquelle F., Cardona N.I., Gómez-Marín J.E., Betbeder D. (2025). T-cell activation of *Toxoplasma gondii* positive donors by maltodextrin nanoparticles formulated with killed *Toxoplasma gondii*. BMC Infect. Dis..

[56] Fasquelle F., Vreulx A.C., Betbeder D. (2024). Improved ELISPOT protocol for monitoring Th1/Th17 T-cell response following *T. gondii* infection. PLoS ONE.

[57] Lee W.H., Kim E. (2026). Mucosal Vaccine Development: From Adjuvant Design to Next-Generation Delivery Strategies. Biomedicines.

[58] An X., Martinez-Paniagua M., Rezvan A., Sefat S.R., Fathi M., Singh S., Biswas S., Pourpak M., Yee C., Liu X. (2021). Single-dose intranasal vaccination elicits systemic and mucosal immunity against SARS-CoV-2. iScience.

[59] Sana M., Rashid M., Rashid I., Akbar H., Gomez-Marin J.E., Dimier-Poisson I. (2022). Immune response against toxoplasmosis—Some recent updates RH: *Toxoplasma gondii* immune response. Int. J. Immunopathol. Pharmacol..

[60] Murillo-Léon M., Bastidas-Quintero A.M., Steinfeldt T. (2024). Decoding *Toxoplasma gondii* virulence: The mechanisms of IRG protein inactivation. Trends Parasitol..

[61] Butcher B.A., Fox B.A., Rommereim L.M., Kim S.G., Maurer K.J., Yarovinsky F., Herbert D.R., Bzik D.J., Denkers E.Y. (2011). *Toxoplasma gondii* Rhoptry Kinase ROP16 Activates STAT3 and STAT6 Resulting in Cytokine Inhibition and Arginase-1-Dependent Growth Control. PLoS Pathog..

[62] Sangaré L.O., Yang N., Konstantinou E.K., Lu D., Mukhopadhyay D., Young L.H., Saeij J.P.J. (2019). Toxoplasma GRA15 Activates the NF-κB Pathway through Interactions with TNF Receptor-Associated Factors. mBio.

[63] Rezaei F., Sharif M., Sarvi S., Hejazi S.H., Aghayan S., Pagheh A.S., Dodangeh S., Daryani A. (2019). A systematic review on the role of GRA proteins of *Toxoplasma gondii* in host immunization. J. Microbiol. Methods.

[64] Liu Z., Yin L., Li Y., Yuan F., Zhang X., Ma J., Liu H., Wang Y., Zheng K., Cao J. (2016). Intranasal immunization with recombinant *Toxoplasma gondii* actin depolymerizing factor confers protective efficacy against toxoplasmosis in mice. BMC Immunol..

[65] Sanchez S.G., Besteiro S. (2021). The pathogenicity and virulence of *Toxoplasma gondii*. Virulence.

[66] Behnke M.S., Khan A., Lauron E.J., Jimah J.R., Wang Q., Tolia N.H., Sibley L.D. (2015). Rhoptry Proteins ROP5 and ROP18 Are Major Murine Virulence Factors in Genetically Divergent South American Strains of *Toxoplasma gondii*. PLoS Genet..

[67] Farhab M., Aziz M.W., Shaukat A., Cao M.-X., Hou Z., Huang S.-Y., Li L., Yuan Y.-G. (2025). Review of Toxoplasmosis: What We Still Need to Do. Vet. Sci..

[68] Ducournau C., Moiré N., Carpentier R., Cantin P., Herkt C., Lantier I., Betbeder D., Dimier-Poisson I. (2020). Effective Nanoparticle-Based Nasal Vaccine Against Latent and Congenital Toxoplasmosis in Sheep. Front. Immunol..

[69] Fasquelle F. (2022). Effective Immuno-Therapeutic Treatment of Canine Leishmaniasis. https://osf.io/8qwh4/overview.

